# Rapidly progressive obstructive bioprosthetic valve thrombosis after surgical aortic valve replacement: a case report

**DOI:** 10.1093/ehjcr/ytaf289

**Published:** 2025-06-20

**Authors:** Shin Hasegawa, Soh Hosoba, Takeshi Mori, Akimitsu Tanaka, Takeki Ohashi

**Affiliations:** Department of Cardiology, Nagoya Tokushukai General Hospital, 2-52 Kozojicho Kita, Kasugai, Aichi 487-0016, Japan; Department of Cardiovascular Surgery, Nagoya Tokushukai General Hospital, 2-52 Kozojicho Kita, Kasugai, Aichi 487-0016, Japan; Department of Cardiology, Nagoya Tokushukai General Hospital, 2-52 Kozojicho Kita, Kasugai, Aichi 487-0016, Japan; Department of Cardiology, Nagoya Tokushukai General Hospital, 2-52 Kozojicho Kita, Kasugai, Aichi 487-0016, Japan; Department of Cardiovascular Surgery, Nagoya Tokushukai General Hospital, 2-52 Kozojicho Kita, Kasugai, Aichi 487-0016, Japan

**Keywords:** Bioprosthetic valve thrombosis, Aortic valve replacement, Cardiac arrest, Case report, Extracorporeal membrane oxygenation

## Abstract

**Background:**

Bioprosthetic valve thrombosis (BPVT) is an uncommon but potentially life-threatening complication following aortic valve replacement. The optimal management approach, whether surgical intervention or thrombolytic therapy, is controversial.

**Case summary:**

A 65-year-old male presented with exertional dyspnoea, and echocardiography confirmed severe aortic valve stenosis. Following minimally invasive aortic valve replacement with a bioprosthetic valve, the patient was discharged on postoperative Day 7 without complications. However, he returned on post-operative Day 113 with acute chest pain and dyspnoea, resulting in cardiac arrest that required extracorporeal cardiopulmonary resuscitation. Subsequent transoesophageal echocardiography revealed significant thrombus formation on the bioprosthetic valve, leading to reoperation and implantation of a new bioprosthetic valve.

**Discussion:**

This case illustrates the rapid progression to obstructive BPVT following discontinuation of anticoagulation therapy. It underscores the potential risk of BPVT despite appropriate anticoagulation. The incidence of BPVT may be underestimated, highlighting the necessity for further investigation into associated risk factors and preventive strategies. Acknowledging the unusual occurrence of BPVT, prompt diagnosis with echocardiographic screening and vigilant follow-up are essential to prevent severe complications such as rapid clinical deterioration and cardiac arrest.

Learning points

- Obstructive bioprosthetic valve thrombosis (BPVT) can develop early after aortic valve replacement (AVR), even with adequate anticoagulation therapy.
- Early obstructive BPVT can lead to severe clinical consequences including sudden cardiac deterioration.
- This case illustrates the importance of vigilance and timely management in patients at risk of BPVT following AVR.

## Introduction

Bioprosthetic valve thrombosis (BPVT) is a rare occurrence, and the decision regarding surgical intervention between thrombolytic therapies remains controversial.^1–3^ Despite appropriate anticoagulation therapy following surgical AVR, there are no reports of rapid progression to obstructive BPVT after discontinuation of warfarin.

## Summary figure

**Figure:**
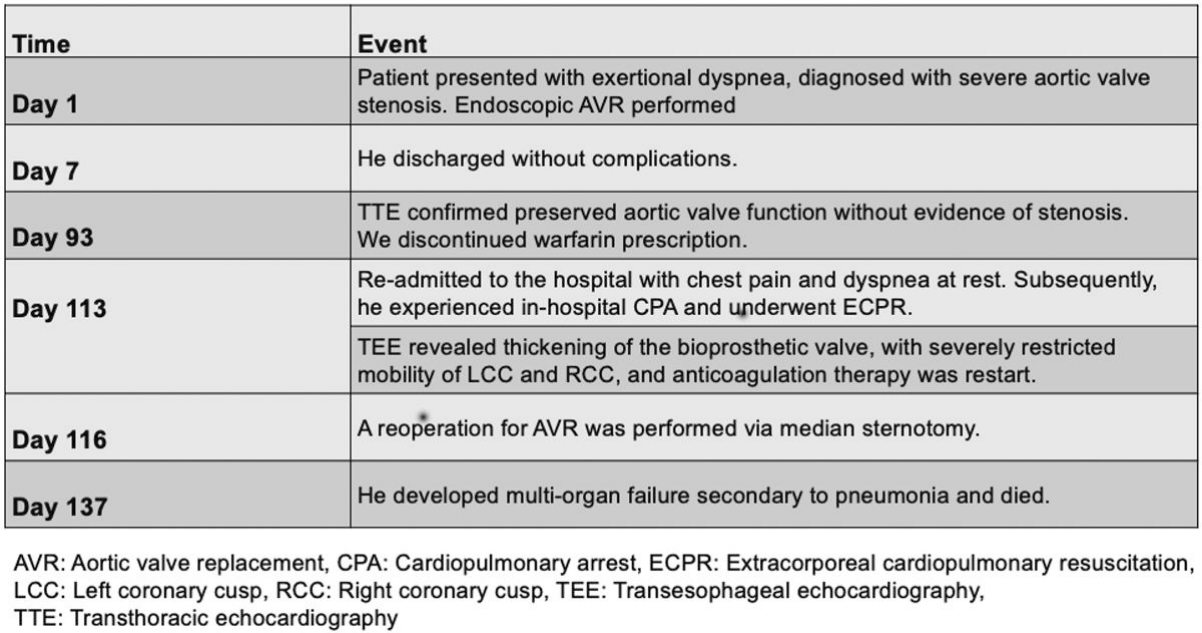


## Case presentation

A 65-year-old male presented with exertional dyspnoea. A chest X-ray revealed cardiomegaly, while echocardiography demonstrated severe aortic valve stenosis (AS). His medical history was notable for alcoholic liver disease, gout, chronic kidney disease, and prostate cancer, which had been managed with endocrine therapy 2 years prior. Minimally invasive aortic valve replacement (AVR) was performed utilizing a 25 mm bioprosthetic valve (Epic, Abbott Laboratories, IL, USA). Our standard regimen of aspirin 100 mg and warfarin was initiated post-operative Day 1. On post-operative Day 7, the patient was discharged without complications. A transthoracic echocardiography (TTE) on post-operative Day 93 showed a left ventricular ejection fraction (LVEF) of 63%, an effective orifice area index of 0.84 cm²/m², and a mean pressure gradient (MPG) of 10 mmHg, confirming preserved bioprosthetic valve function. Warfarin was subsequently discontinued.

**Figure 1:**
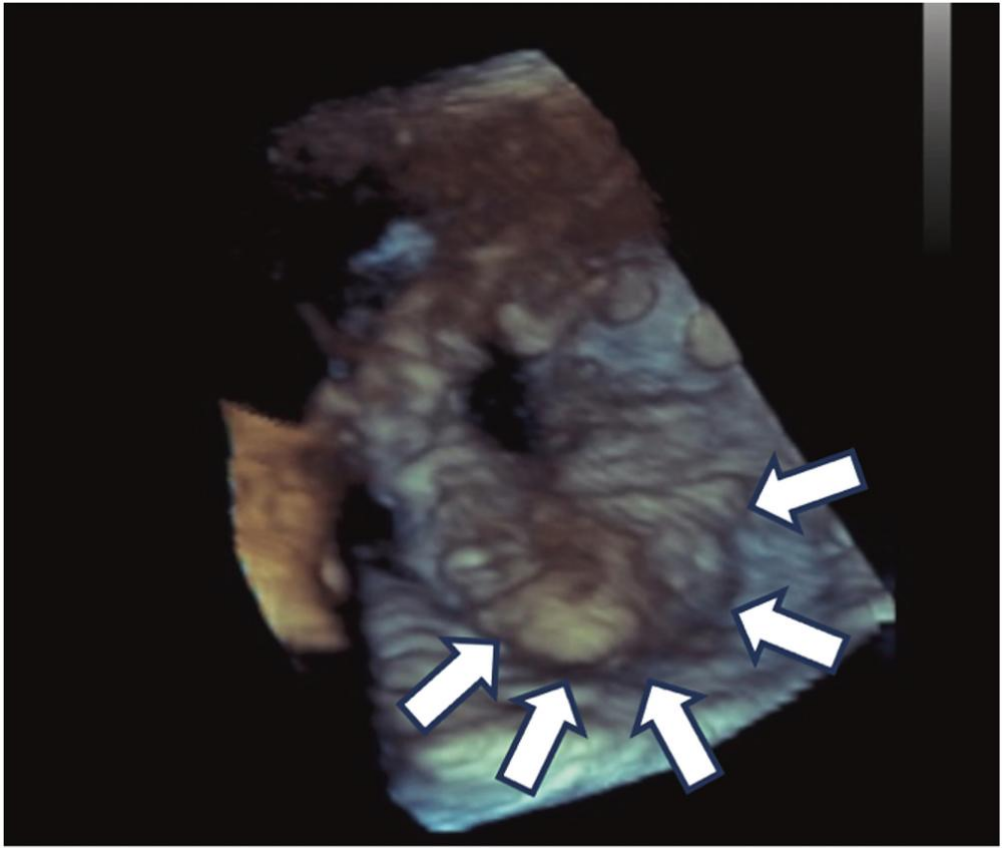
Transoesophageal echocardiography of obstructive bioprosthetic valve thrombosis. 3D echocardiographic images of the bioprosthetic valve after surgical aortic valve replacement. A thrombus is present on the prosthetic valve, restricting its motion (white arrows).

**Figure 2:**
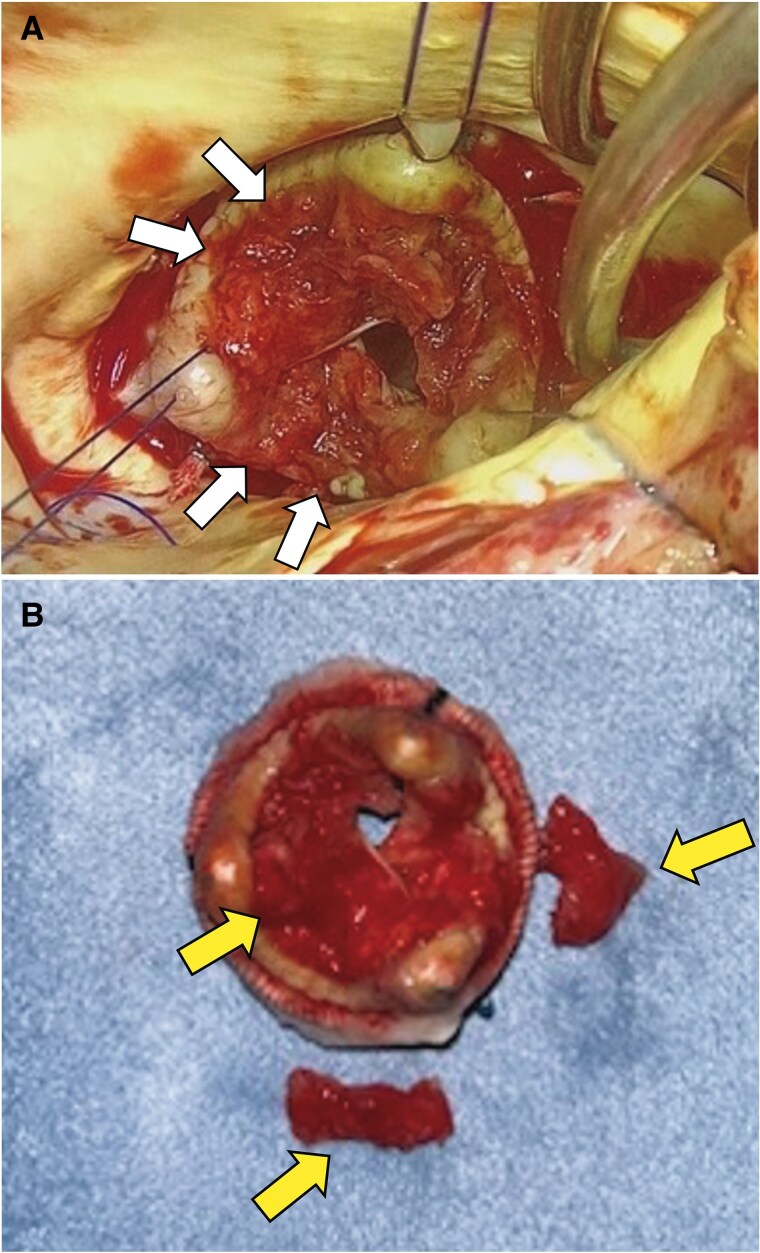
Bioprosthetic valve thrombosis after surgical aortic valve replacement. 3D scope demonstrates significant bioprosthetic valve thrombosis. Two of the three leaflets of the prosthetic valve are immobile (arrows in A). An extensive thrombus (arrows in B) was adherent to the bioprosthetic valve.

On post-operative Day 113 (20 days following warfarin discontinuation), the patient returned with chest pain and dyspnoea at rest. He exhibited hypotension, with a blood pressure of 80/59 mmHg, a heart rate of 115 b.p.m., and a respiratory rate of 30/min. Physical examination revealed peripheral coldness and cyanosis. Pulmonary auscultation demonstrated bilateral crackles. The patient experienced in-hospital cardiopulmonary arrest (CPA) and underwent extracorporeal cardiopulmonary resuscitation (ECPR). Electrocardiography demonstrated normal sinus rhythm with a heart rate of 100 b.p.m., along with newly observed T wave inversions in precordial leads V3–6. A chest X-ray demonstrated bilateral pulmonary oedema and cardiomegaly, and coronary angiography revealed no significant coronary artery disease. The MPG across the aortic valve was 39 mmHg, and left ventricular function was diminished. Transthoracic echocardiography indicated a reduced LVEF of 34% (Supplementary material online, *Video TTE 01*). Transoesophageal echocardiography (TEE) confirmed severe bioprosthetic AS and moderate regurgitation with significant echodensity on the valve leaflets, suggesting restricted leaflet mobility and raising concerns for valve thrombosis (*Figure 1*) (Supplementary material online, *Video TEE 01-04*). Laboratory investigations revealed a markedly elevated B-type natriuretic peptide level of 4310 pg/mL, measured during the episode of cardiac arrest. A complete blood count showed thrombocytopenia, associated with reduced protein S levels and decreased protein C activity, likely due to the low platelet count. No evidence suggestive of an autoimmune disease was found. Cardiothoracic surgery was consulted for the potential reoperation of surgical AVR. However, the patient was deemed extreme risk due to multiple factors, including impaired cardiac function in mechanical support and a history of previous cardiac surgery. Our heart team discussed management strategies with the patient, including the use of extracorporeal membrane oxygenation (ECMO) and Impella CP (Abiomed, Danvers, MA, USA) for heart failure secondary to obstructive BPVT. Subsequent computed tomography imaging revealed severe pulmonary congestion. Despite attempts to maintain the activated clotting time within the target range of 180–200 s using intravenous unfractionated heparin, no significant improvement in valve function was observed. Following the resolution of the patient’s pulmonary oedema and the subsequent improvement in the ventilator requirement, and on hospital Day 4, a reoperation for AVR was performed via median sternotomy. Intraoperative second-look TEE did not show any improvement in valve function. Thrombus formation was observed on the explanted bioprosthetic valve at all three leaflets, especially severe on the left coronary cusp and right coronary cusp (*Figure 2A* and *B*). A new 25 mm bioprosthetic valve (Inspiris Resilia, Edwards Lifesciences LLC, CA, USA) was implanted.

Following redo AVR, the patient experienced persistent hypotension that required continued management with inotropes and ECMO post-operatively. On post-operative Day 7, hypotension developed due to infection and bleeding from the wound and alveoli, necessitating additional mechanical circulatory support. Due to the patient’s bleeding tendency, Impella was avoided, and an intra-aortic balloon pump (IABP) was selected for haemodynamic support. Extracorporeal membrane oxygenation was gradually weaned by post-operative Day 10, and the IABP was removed on post-operative Day 15. However, the patient developed multi-organ failure secondary to pneumonia and died on post-operative Day 25.

## Discussion

Bioprosthetic valve thrombosis can be attributed to various factors, including atrial fibrillation, interruption of anticoagulation therapy, a history of thromboembolism, diminished cardiac function, and known coagulopathies.^4,5^ The pathophysiology of prosthetic valve thrombosis (PVT) is complex and multifactorial, involving interactions among valve components, blood flow dynamics through prosthetic conduits, and underlying hypercoagulable states.^6^ The incidence of BPVT in aortic position has been reported to range from 0.03% to 1.26%.^1,2^ However, Egbe *et al*.^7^ indicated that among 397 patients who underwent reoperation following valve replacement, 46 cases (11.6%) were attributed to BPVT, suggesting that this condition is more prevalent than previously acknowledged.

In this case, anticoagulation therapy with warfarin was administered for 3 months, and TTE confirmed preserved aortic valve function without evidence of stenosis. However, 20 days after the discontinuation of warfarin, the patient developed obstructive BPVT, leading to cardiogenic shock classified as Stage C by Society for Cardiovascular Angiography and Interventions, rapidly progressing to Stage D, and ultimately requiring ECPR following in-hospital CPA.^8^ Existing literature details early PVT of pure carbon bi-leaflet aortic valves and early obstructive BPVT following transcatheter aortic valve-in-valve procedures.^6,9^ This case involved surgical AVR with a bioprosthetic valve, and there are no prior reports of rapid deterioration culminating in CPA after appropriate anticoagulation therapy. The optimal antithrombotic strategy in the early post-operative period following surgical implantation of a bioprosthetic valve remains controversial. Multiple observational studies support the use of warfarin to reduce the risk of thromboembolism.^10^ However, prior studies have also reported the occurrence of a rebound hypercoagulable state following the discontinuation of warfarin, which may have contributed to the formation of thrombosis in the present case.^11^

Management of obstructive BPVT may necessitate thrombolytic therapy and surgical intervention.^1,12^ In this instance, due to haemodynamic instability, heart failure management and anticoagulation therapy were implemented, with surgical intervention selected following improvement in the haemodynamic status. Future larger studies are essential to investigate the incidence and risk factors associated with obstructive BPVT. Furthermore, consideration should be given to the potential for a rapid decline in aortic valve mobility following surgical AVR with bioprosthetic valves. Given the discontinuation of anticoagulation therapy, short-term follow-up may be necessary for patients with bioprosthetic valves.

This case underscores the incidence of CPA attributable to obstructive BPVT following AVR. It emphasizes the necessity to consider the potential for rapid progression to obstructive BPVT, even after a suitable duration of anticoagulation therapy, as the cessation of warfarin may precipitate a critical deterioration in valve function. Obstructive BPVT can result in severe heart failure, thereby necessitating a meticulous evaluation of the risks associated with complications of bioprosthetic valves in the early post-operative period.

## Lead author biography


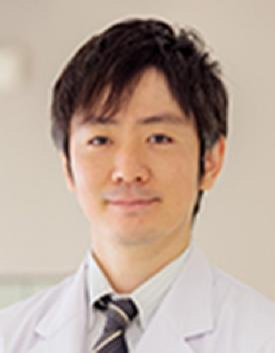


Shin Hasegawa graduated from Gifu University School of Medicine in 2015 and completed his initial training there until 2017. Since 2017, he has been working in the Department of Cardiology at Nagoya Tokushukai General Hospital. His interests within cardiovascular medicine include the treatment of structural heart disease, arrhythmias, and aortic diseases.

## Supplementary Material

ytaf289_Supplementary_Data

## Data Availability

The data that support the findings of this study are available from the corresponding author upon reasonable request. No restricted data were used in this study.

## Supplementary material

**Consent:** Informed consent for the publication of this case report and accompanying images was obtained from the patient and his relatives. The publication adheres to institutional ethics guidelines, and no personal identifiers were included to ensure the patient’s privacy and confidentiality. The study was approved by the Institutional Review Board.

**Funding**: None declared.
